# Association of low serum TGF-β level in hantavirus infected patients with severe disease

**DOI:** 10.1186/s12865-015-0085-0

**Published:** 2015-04-14

**Authors:** Mahmoud Sadeghi, Imad Lahdou, Jakob Ettinger, Mojdeh Heidary Navid, Volker Daniel, Martin Zeier, Jörg Hofmann, Gerhard Opelz, Paul Schnitzler

**Affiliations:** Department of Transplantation Immunology, University of Heidelberg, Im Neuenheimer Feld 305, 69120 Heidelberg, Germany; Institute of Medical Virology, Charité University Medicine Berlin, Charitéplatz 1, 10117 Berlin, Germany; Department of Infectious Diseases, Virology, University of Heidelberg, Im Neuenheimer Feld 324, 69120 Heidelberg, Germany; Department of Nephrology, University of Heidelberg, Im Neuenheimer Feld 162, 69120 Heidelberg, Germany

**Keywords:** Hantavirus, Puumala, Transforming growth factor, TGF, Severe disease

## Abstract

**Background:**

Hantaviruses are emerging zoonotic pathogens which cause hemorrhagic fever with renal syndrome, an immune-mediated pathogenesis is discussed. The aim of the present study was to investigate the role of TGF-β expression in acute hantavirus infection.

**Results:**

We retrospectively studied 77 patients hospitalised with acute *Puumala* infection during a hantavirus epidemic in Germany in 2012. Hantavirus infection was confirmed by positive anti-Puumala hantavirus IgG and IgM. Plasma levels of transforming growth factor (TGF)-β1 and TGF-β2 were analysed. Based on glomerular filtration rate on admission, patients were divided in mild and severe course of disease. Puumala virus RNA was detected by PCR amplification of the viral L segment gene. Out of 77 Puumala virus infected patients, 52 (68%) were male. A seasonal distribution was detected in our cohort with a peak in summer 2012, the highest incidence was observed in the age group of 30–39 years. Puumala virus RNA was detectable in 4/77 cases. Patients with severe disease had a significant longer hospital stay than patients with mild disease (6.2 vs 3.6 days). Thrombocyte count (186 vs 225 per nl), serum TGF-β1 (74 vs 118 ng/l) and TGF-β2 (479 vs 586 pg/l) were significantly lower in severe compared to mild disease. However, C-reactive protein (CRP) was significantly higher in patients with severe disease (62 vs 40 mg/l). TGF-β1/Cr was the most sensitive and specific marker associated with renal dysfunction.

**Conclusion:**

High serum CRP and low serum TGF-β in the early phase of hantavirus infection is associated with a severe course of disease. Our results support the hypothesis of an immune-mediated pathogenesis in hantavirus infection.

## Background

Three main clinical syndromes can be distinguished after hantavirus infection, i.e. hemorrhagic fever with renal syndrome (HFRS) mainly caused by Seoul, Puumala and Dobrava viruses; nephropathia epidemica, a mild form of HFRS caused by Puumala virus; and hantavirus cardiopulmonary syndrome, which may be caused by Andes virus and Sin Nombre virus. There is no curative treatment for hantavirus infection, and eliminating or minimising contact with rodents is the best way to prevent infection [1]. The case fatality rate of HFRS varies from <1% to 12% depending on the virus type [2]. Over 50 000 nephropathia epidemica cases with Puumala virus infections have been registered in Europe, and even over 175 000 in Western Russia [3]. Approximately 150,000 to 200,000 humans are hospitalized each year because of hantavirus infections worldwide [2]. European hantaviruses of the family *Bunyaviridae* cause HFRS. Infection in humans occurs through inhalation of aerosolised virus particles from excreta of chronically infected wild rodents [4]. Puumala is reported throughout most of Europe (excluding the Mediterranean region) wheras Dobrava, carried by the yellow-necked mouse (*Apodemus flavicollis*) and Saaremaa, carried by the striped field mouse (*Apodemus agrarius*), are reported mainly in eastern and central Europe [5]. In Germany, several hantaviruses pathogenic for humans are circulating, i.e. Puumala virus, Dobrava virus and Tula virus [6,7]. Puumala virus is carried by the bank vole (*Clethrionomys glareolus*) and leads mostly to a mild form of disease [3].

The course of HFRS is highly variable, ranging from frequently asymptomatic to a lethal outcome. Host genetic factors influence the clinical outcome. The most common symptoms are high fever, headache, abdominal pain, backache and nausea or vomiting. Proteinuria, haematuria and acute kidney injury are signs of renal involvement. Classically, HFRS occurs in five distinct phases: febrile, hypotensive, oliguric, polyuric and convalescent [8]. A severe course of disease comprises oliguria, high blood creatinine and a high leukocyte count [9], the disease severity depends on the hantavirus genotype. A minority of patients needs transient dialysis treatment, but complete recovery is the usual outcome [10,11]. Outinen et al. defined the severity of hantavirus infection by serum creatinine and thrombocytes count [12]. The role of immune response and cytokine expression during Puumala virus infection has been described previously [13,14]. Techniques for the identification of novel hantaviruses by specific cell culture models had been established [15]. Only few cases of Puumala virus infection with severe disease had been reported for Germany recently [16,17].

In the year 2012, the highest number of human hantavirus infections were observed and more than 2800 cases were reported, 30% of these infections were located in Southwestern Germany. Case numbers started to rise earlier during the year than had been reported in previous epidemics in 2007 and 2010, and are the largest ever reported in this region [18,19]. The early rise might be associated with a birch mast year in 2011, followed by an early and massive reproduction of the reservoir of bank vole populations during winter 2011 and spring 2012 [20]. However, a possible influence of climate change on the survival, emergence and epidemiology of hantaviruses is difficult to predict [21].

It has been suggested that viral load and immunological factors including cytokines are involved in the pathogenesis of Puumala virus infection. The extent and level of viremia depends largely on the hantavirus type, viral load is considerably lower in HFRS caused by Puumala virus when compared to other hantaviruses. Thus viral load clearly plays a role in disease pathogenesis [8]. In a recent study, patients infected with Dobrava virus were found to have a higher viral load than Puumala infected patients [13]. The aim of this study was to analyse TGF-ß serum levels in patients with hantavirus infection and to evaluate the level of TGF-ß in mild and severe course of disease.

## Results

### Patient characteristics and clinical findings

**Figure 1:**
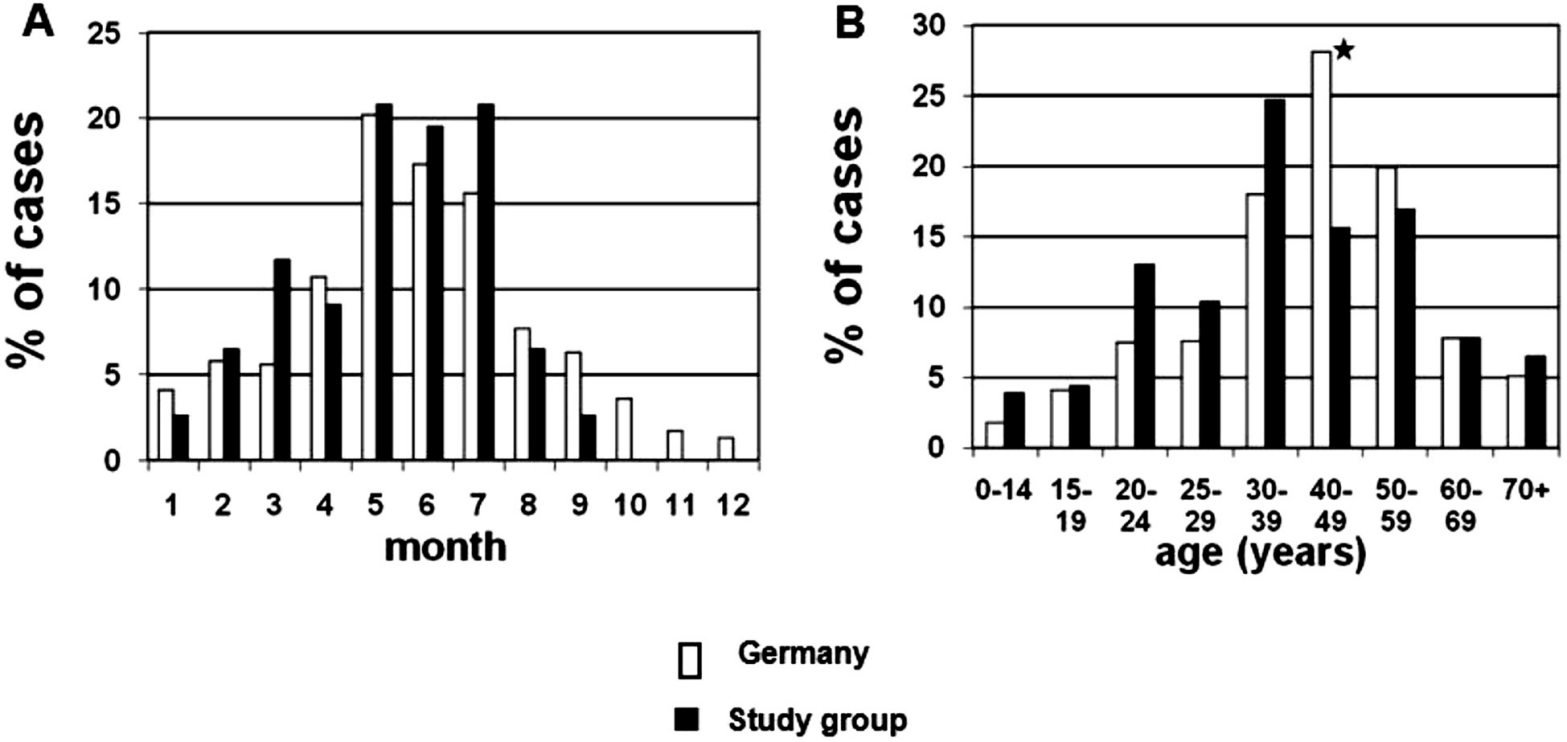
**Seasonality (A) and age distribution (B) of hantavirus infections in Germany (white bars) and our study cohort (black bars).**

On admission, all study patients were seropositive for anti-Puumala IgG and IgM antibodies. Out of 77 patients with acute Puumala virus infection, 52 (68%) were male. A seasonal distribution of hantavirus infection was detected in our cohort and for nationwide cases with a peak in summer 2012. Age distribution showed a higher percentage of patients in the age groups of 20–39 (48.1% vs 33.1%; p < 0.05) years and a lower percentage of patients in the age groups of 40–59 (32.5% vs 48.0%; p < 0.05) years in our cohort compared to nationwide data (Figure 1). In contrast to the highest incidence in the age group of 40–49 (28.1% vs 15.6%; p < 0.05) years nationwide, our study patients demonstrated the highest proportion in the age group of 30–39 years (24.7% vs 18.0%; p > 0.05, not significant).

For all patients mean hospitalization time was 4.9 days. On admission, demographic data and clinical symptoms such as fever, myalgia, and headache were similar in patients with mild and severe disease (Table 1). However, patients with a severe course of disease defined by a glomerular filtration rate (GFR) of <30 ml/min had significantly longer hospital stay (6.2 vs 3.6 days) and significantly higher proteinuria levels (1.3 vs 0.6 g/l) compared to patients with mild disease, respectively. The most common symptoms reported on admission were myalgia, fever, headache, shivering, low back pain, and arthralgia (Table 1). All patients had impaired renal function to some degree on admission with an increase in retention parameters over the course of disease, six patients (8%) required intermittent hemodialysis.

**Table 1:** **Clinical data of Puumala virus infected patients with mild and severe disease on admission**

**Parameters**	**Mild disease (n = 38)**	**Severe disease (n = 39)**	**p-value**
age in years (mean ± SD)	39.2 ± 15.5	42.7 ± 16.9	0.34
male	63.2%	71.8%	0.42
hospital stay (days ± SD)	3.6 ± 3.6	6.2 ± 3.4	0.001
myalgia	87%	87%	0.69
fever	84%	82%	0.80
headache	84%	82%	0.80
shivering	82%	80%	0.82
low back pain	82%	74%	0.45
arthralgia	71%	87%	0.27
stomach ache	63%	56%	0.74
nausea	58%	54%	0.72
vomiting	42%	33%)	0.43
diarrhea	18%	10%	0.31
vision disturbance	11%	15%	0.53
dyspnoea	13%	10%	0.69
thrombocytes min. (/nl ± SD)	206 ± 131	177 ± 115	0.48
proteinuria (g/l ± SD)	0.6 ± 1.5	1.3 ± 2.3	0.009

Symptoms are shown in % of patients.

### Correlation of cytokine expression with disease severity

**Figure 2:**
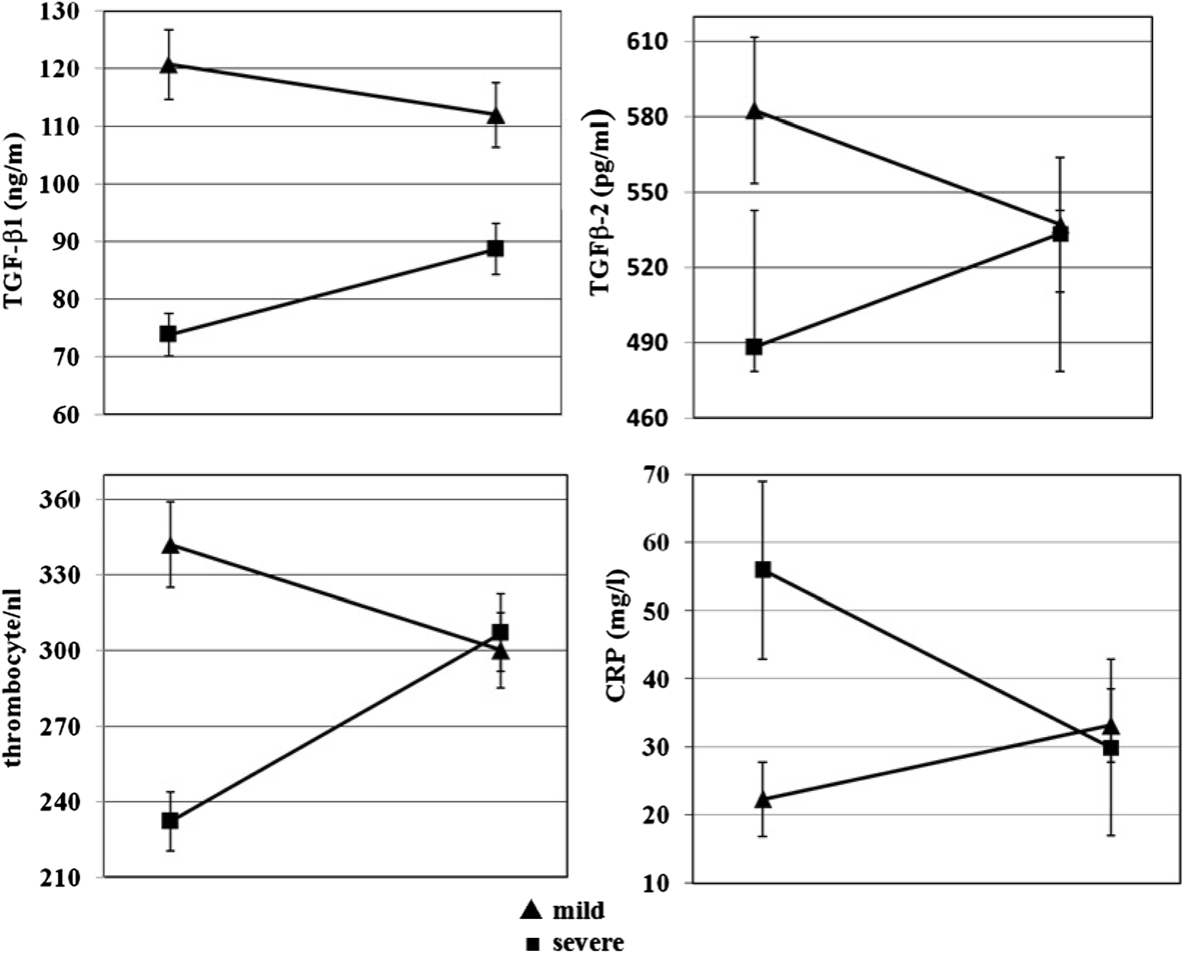
**Serum TGF-β1, TGF-β2 concentration, thrombocyte count and CRP in patients with mild or severe Puumala virus infection. Values are given for the time on admission and one week after admission.**

**Figure 3:**
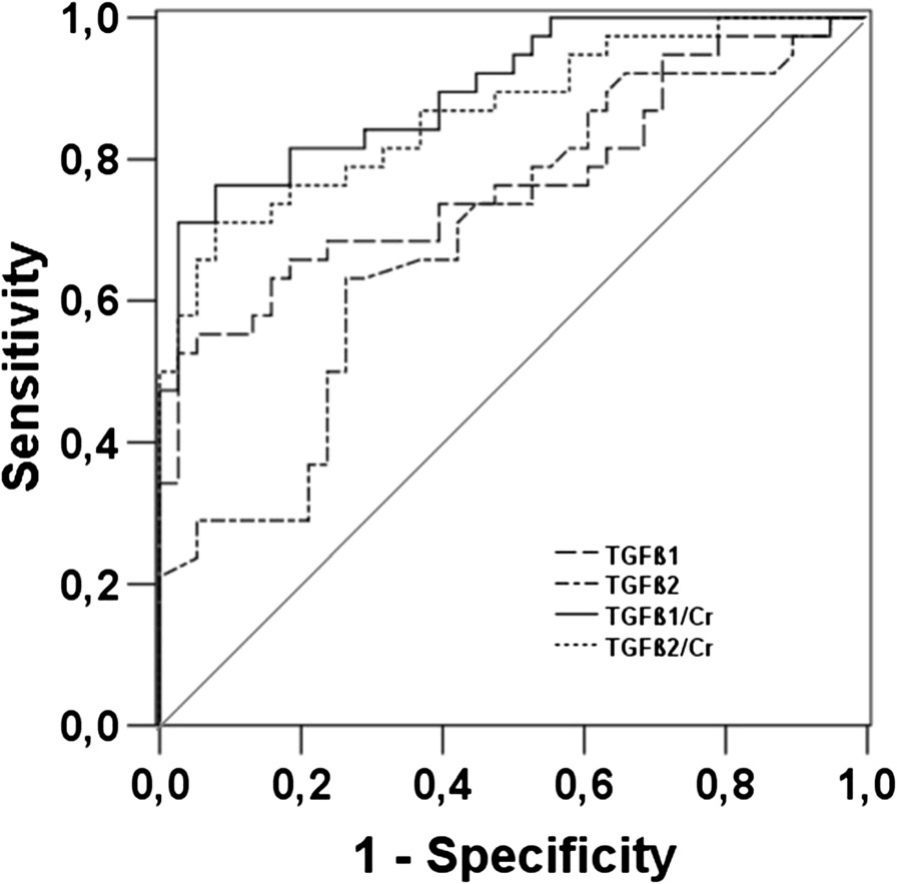
**ROC curve analysis of TGF-β1, TGF-β2, TGF-β1/Cr, and TGF-β2/Cr.**

On admission, CRP was increased (62 vs 40 mg/l), a similar finding was observed for creatinine (5.6 vs. 2.4) in patients with severe and mild disease, respectively. Interestingly, patients with severe disease had significantly lower TGF-β1 and TGF-β2 serum levels than patients with mild disease, suggesting an association of TGF-ß1 and TGF-ß2 with disease severity (Table 2). All patients had non-detectable TGF-β3 serum levels. The course of cytokine levels for TGF-ß1, TGF-ß2, as well as CRP and thrombocyte count on admission and one week after admission to the hospital is shown in Figure 2 and Table 3. TGF-ß1 and TGF-ß2 concentrations on admission imply a stronger TGF-ß1 and TGF-ß2 production in patients with severe than in patients with mild disease (Figure 2). Both patient groups had similar CRP level and thrombocyte count one week after admission. Creatinine remained significantly higher in patients with severe disease during follow-up (data not shown). ROC curve analysis was performed for TGF-β1, TGF-β2 as well as for TGF-β1/Cr and TGF-β2/Cr ratios on admission. TGF-β1/Cr ratio showed the best association with severe disease, followed by TGF-β1/Cr ratio (Figure 3).

**Table 2:** **Laboratory data and cytokine analysis of patients with mild and severe disease on admission**

**Parameters**	**Mild disease (n = 38)**	**Severe disease (n = 39)**	**p-value**
creatinine (mg/dl)	2.41 ± 1.9	5.6 ± 2.6	<0.0001
leukocyte (/nl)	8.6 ± 2.6	9.6 ± 3.7	0.55
thrombocytes (/nl)	342 ± 156	232 ± 136	<0.0001
CRP (mg/l)	40 ± 36	62 ± 45	<0.0001
TGF-β1 (ng/ml)	118 ± 47	74 ± 28	0.0001
TGF-β1/Cr	76 ± 60	17 ± 10	<0.0001
TGF-β2 (pg/ml)	586 ± 155	479 ± 146	0.004
TGF-β2/Cr	384 ± 283	110 ± 68	<0.0001
sCD30 (U/ml)	104 ± 63	126 ± 71	0.17
sCD30/Cr	60 ± 48	29 ± 29	0.0002
neopterin (nmol/l)	172 ± 246	186 ± 227	0.41
neopterin/Cr	70 ± 99	36 ± 37	0.04

**Table 3:** **Mean and median serum values of TGF-β1, TGF-β2, and TGF-β/Cr ratios**

**TGF-β**	**Acute phase (n = 39)**	**One week follow-up (n = 29)**	**p-value**
TGF-β1 (ng/ml; mean ± SD)	74 ± 28	89 ± 39	0.020
TGF-β1 (ng/ml; median)	74	86	0.020
TGF-β2 (pg/ml; mean ± SD)	479 ± 146	533 ± 118	0.008
TGF-β2 (pg/ml; median)	488	534	0.008
TGF-β1/Cr (mean ± SD)	17 ± 10	37 ± 40	0.020
TGF-β1/Cr (median)	13	22	0.020
TGF-β2/Cr (mean ± SD)	110 ± 68	212 ± 197	0.008
TGF-β2/Cr (median)	88	117	0.008

Cr: creatinine

### Phylogenetic analysis of puumala virus

**Figure 4:**
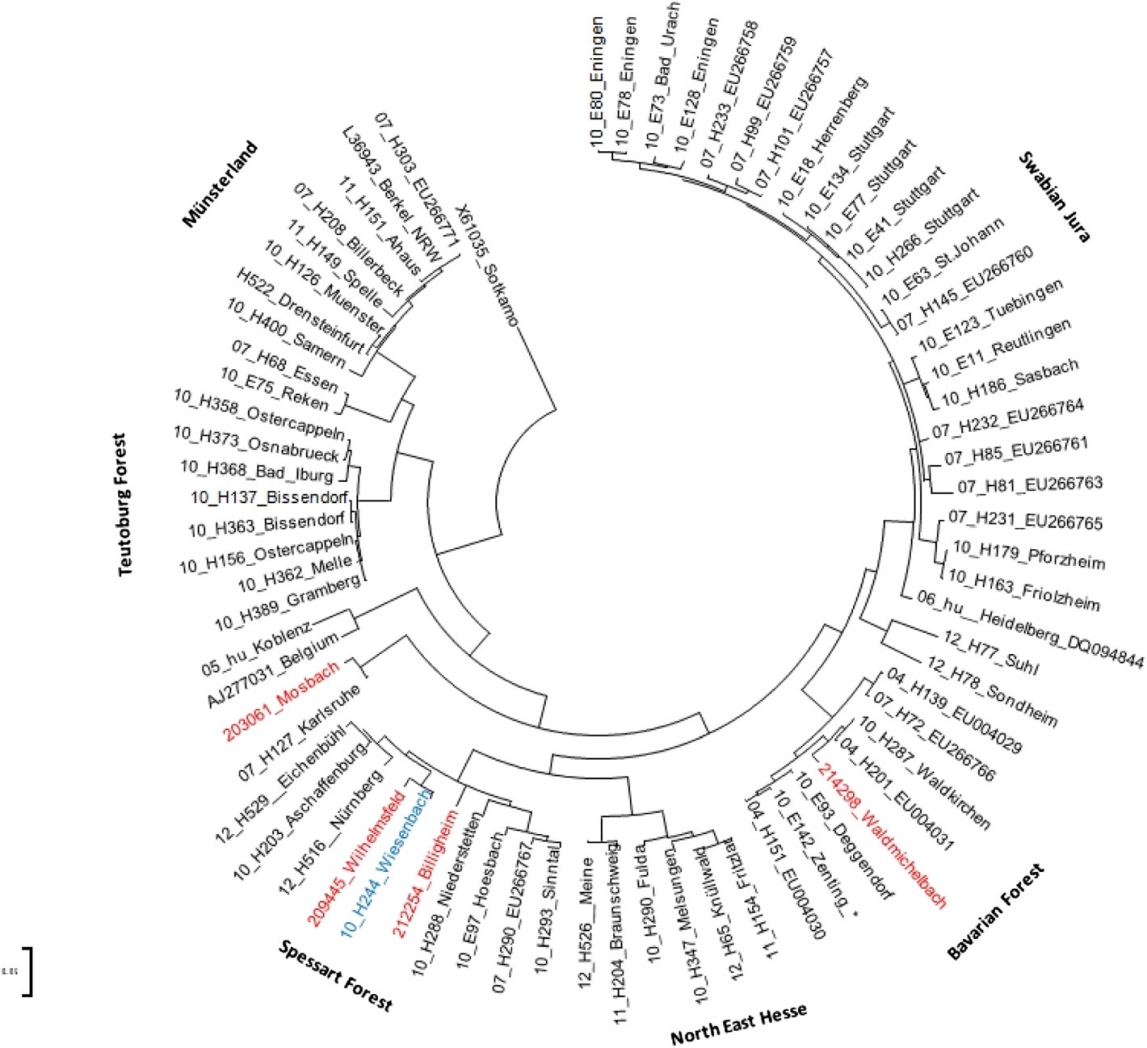
**Neighbor-joining phylogenetic tree (TN93 evolutionary model) of human Puumala virus (PUUV) strains based on partial sequences of the S segment (504 nt, position 392–894).** PUUV-sequence from strain Sotkamo was used as an outgroup. Sequences taken from GenBank are indicated by their accession numbers. The numbers 04 to 12 in front of the sample names indicate the year (2004–2012) when the sample was collected. Four sequences obtained in this study are colored in red (acc.-no.: KM013472-KM013475) and an additional sample (in blue) was published in an earlier study [Reference PMID: 22958255]. Phylogenetic clades are described earlier [Reference PMID: 22932394]. Scale bar indicates an evolutionary distance of 0.1 substitutions per position.

For phylogenetic analysis, samples positive for hantavirus RNA were sequenced in the hantavirus S-segment. The data revealed no unique hantavirus strain in these patients. The sequence data indicate a heterogeneous strain reservoir in Southwest Germany, some hantavirus strains showed the highest relatedness to strains from the Spessart forest or the Bavarian forest (Figure 4).

## Discussion

Pathogenesis of hantavirus is not well understood, but most likely virus-mediated, host-mediated mechanisms as well as viral load are involved [22]. Most European and Asian cases of hantavirus infection are characterized by acute renal failure, thrombocytopenia and increased vascular permeability [19]. Acute renal failure is observed in 90–95% of infections with Old World hantaviruses. The long-term prognosis of nephropathia epidemica (NE) caused by Puumala virus is favorable and most patients fully recover renal function [19].

Several previous studies reported an association of pro-inflammatory cytokines and severity of hantavirus infections [14,23-26]. There are only few reports about the role of anti-inflammatory and suppressive cytokines such as TGF-β in hantavirus infection. Temonen et al. investigated kidney biopsies of patients with nephropathia epidemica and described an increased expression of the cytokines TNF-α, TGF-β, and platelet-derived growth factormainly in the peritubular area of the distal nephron. Concomitantly also at the same location expression of the endothelial adhesion molecules ICAM-1, VCAM, and PECAM was seen [27]. Similarly, it was shown in a more recent study of deer mice infected with Sin Nombre virus, that TGF-β1 expressing regulatory T cells may play an important role in limiting immunopathology in the natural reservoir host, but this response may interfere with viral clearance [28]. In a previous study it was shown that disease severity characterized by elevated creatinine and low platelet counts was correlated with high pro-inflammatory IL-6 and TNF-α but low immunosuppressive TGF-β1 levels and vice versa, suggesting the induction of a protective immune mechanism to downregulate or even prevent a massive pro-inflammatory immune response [14]. Klempa et al. described mild and severe cases of hemorrhagic fever with renal syndrome in patients infected with Dobrava-Belgrade virus in Russia [29]. In accordance with our results, they found a high number of patients with headache in severe hantavirus infection and elevated creatinine in severe course of the disease. In the current study, we were able to illustrate the role of TGF-β1 and TGF-ß2 as major protective markers of hantavirus infection. With increased production in patients with severe disease as a response to inflammation [30,31], TGF-β1 and TGF-β2 serum levels reached similar levels in both patient groups one week after admission. Obviously, patients with severe disease generate TGF-ß delayed compared to patients with mild disease allowing stronger proinflammatory cytokine responses and inflammation in these patients.

TGF-β has a pivotal function in the immune system and controls the initiation and resolution of inflammatory responses through the regulation of chemotaxis, activation, and survival of lymphocytes, natural killer cells, dendritic cells, macrophages, mast cells, and granulocytes [32]. There are reports of increased TGF-β during many viral infections, such as influenza, HIV, hepatitis B virus and hepatitis C virus infection. The neuraminidase glycoprotein of influenza A and B viruses have been shown to directly activate latent TGF-β [32]. Prognostic and predictive values of TGF-β in acute viral infections have not been studied.

In a macaques model of hantavirus infection, Sironen et al. showed that viral RNA was observed in kidney, spleen and liver tissues. Inflammatory cell infiltrations and tubular damage were found in the kidneys, and these infiltrations contained mainly CD8 T-cells [33]. As reported by Rook et al., TGF-β exerts profound inhibitory effects on T and B lymphocyte proliferation [34]. T regulatory (Treg)- and T suppressor (Ts)-mediated immunosuppression appears to depend on two pathways: cell–cell contact and cytokine secretion, whereby cytokine secretion is assumed to be more important. Intracellular cytokines, such as interferon (IFN)-γ, IL-10 and TGF-β are characteristic for certain Treg subsets and are related with induced Treg function. Stimulation by mitogens significantly induce TGF-β^+^-Treg and -Ts. Tregs are an important component in regulating the magnitude of the immune response to infection, thus preventing excessive inflammation and tissue damage [35]. In previous studies it was suggested that IFN-γ-secreting Treg are forming the first line of immunoregulatory T cells during an initiated immune response, followed by IL-2^−^, IL-10^+^, or TGF-β^+^ Treg. Thus, high serum levels of TGF-βs during the acute phase and convalescence period of hantavirus infection might be a protective parameter against pro-inflammatory cytokines and endothelial injury. We found a positive correlation of both TGF-βs with platelet counts and GFR. In contrast to TGF-βs, serum sCD30 and neopterin showed no significant correlation with main symptoms of disease.

Analysis of cytokines responses during the acute phase in our studied patients revealed a significant difference between patients with mild and severe disease. We showed associations of serum TGF-βs and TGF-βs/Cr ratios during the acute phase of hantavirus infection with GFR and disease severity. Severe anti-inflammatory response was associated with relatively mild disease whereas weak reactions were associated with severe renal failure. These data suggest that plasma TGF-βs levels and especially TGF-βs/Cr ratios during the acute phase of hantavirus infections may serve as a determining factor of disease severity that allow prediction of disease severity.

CRP is an acute phase protein, and during acute phase of NE, an increase in the serum CRP concentration is a typical laboratory finding [12]. The known main functions of CRP are complement activation, enhancement of phagocytosis, and induction of cytokine synthesis. Although the CRP level is widely used as an indicator of the severity of the disease in various infections, there are no reports associating high CRP levels with severe disease in NE or other viral infections. Outinen et al. showed that high plasma IL-6 concentrations are associated with a clinically severe acute Puumala hantavirus infection, whereas, high plasma CRP as such does not reflect the severity of the disease [12]. However, in the present study serum CRP was a hallmark of kidney injury.

In order to analyse a relationship of these findings to a distinct hantavirus genotype circulating in the Heidelberg area, phylogenetic analysis of hantavirus strains was performed. The data revealed no unique hantavirus strain in these patients and indicate a heterogeneous strain reservoir in Southwest Germany. Some hantavirus strains showed the highest relatedness to strains from the Spessart forest or the Bavarian forest and none of the sequenced strains was similar to the Heidelberg strain published previously.

## Conclusions

We conclude that measurement of TGF-βs and especially TGF-βs/Cr ratios in the early phase of Puumala hantavirus infection are very sensitive markers of kidney damage and that high expression of TGF-β1 and TGF-β2 are associated with milder courses of hantavirus infection. High CRP is a marker of disease severity in hantavirus infection. Our results support the hypothesis of an immune-mediated pathogenesis of hantavirus infection.

## Methods

### Study population

In this retrospective study, we analyzed clinical and laboratory data of 77 hospitalised patients diagnosed with acute hantavirus infection at the University Hospital Heidelberg. Glomerular filtration rate (GFR) provides the best index of overall kidney function and creatinine concentration is the most widely used parameter for estimation of GFR [8]. Based on GFR, we divided patients into two groups: patients with mild disease (GFR ≥ 30 ml/min) and patients with severe disease (GFR < 30 ml/min). The first blood sample of each patient was obtained on admission to the hospital, on average 7 days after onset of symptoms. An additional blood sample was obtained one week after admission. All samples were analysed for cytokine and lymphocyte, monocyte/macrophage activating and inflammatory marker expression including transforming growth factor (TGF)-β1, −β2, −β3, sCD30 and neopterin levels. The mean age of patients was 40.8 years, 52 were male (68%), five (6%) were under the age of 18 years.

### Detection of hantavirus antibodies and RNA

Sera were tested for hantavirus IgG and IgM antibodies using the *recom*Line hantavirus assay (Mikrogen, Munich, Germany), coated with specific hantavirus nucleocapsid antigens of Puumala virus, Dobrava-Belgrade virus, Hantaan virus and Seoul virus. Patients were included in the study if both, hantavirus IgG and IgM were positive. Positive results of IgG and negative results of IgM for hantavirus may also indicate an infection with mild clinical symptoms. However, for these patients with positive IgG and negative IgM for hantavirus, reference samples were available indicating no acute but past hantavirus infection. Consequently, these patients had been excluded from the study. PCR for the detection of hantavirus RNA was performed on the L-segment [36]. For phylogenetic analysis positive samples were subsequently sequenced in the S-segment [18]. Characteristics of the sequence analysis are given in the legend to the figure.

### Determination of serum cytokines and CRP

TGF-β1, β2 and β3 were measured with the Luminex TGF assay (R&D Systems, Wiesbaden, Germany). To exclude cytokine accumulation due to dysfunction of renal excretion, cytokine/creatinine ratios were calculated. For ease of viewing, TGF-β1 is shown in ng/ml, whereas TGF-β2 and TGF-β3 are shown in pg/ml. Serum neopterin was measured with the Neopterin ELISA kit (Brahms, Berlin, Germany). Based on control measurements in 36 healthy individuals, neopterin concentrations >15 nmol/l is considered above normal range. Sera were tested for serum sCD30 content using a commercially available ELISA kit (Bender MedSystems, Vienna, Austria). All cytokines, neopterin and CRP were determined on admission of the patients to the hospital and one week after admission.

### Statistical analysis

Categorical and continuous variables were analyzed using chi square, Fisher exact and Mann–Whitney-U tests. Statistical analyses were performed with the Statistical Package for the Social Sciences (SPSS, 18.0). After Bonferroni correction, p values ≤0.05 were considered statistically significant.

### Ethical approval

This study was approved by the Ethics Committee of the Faculty of Medicine, University Heidelberg. Written consent was obtained from patients.
